# Past emergent phase of Shatsky Rise deep-marine igneous plateau

**DOI:** 10.1038/s41598-017-15684-z

**Published:** 2017-11-13

**Authors:** Moriaki Yasuhara, Atsushi Ando, Yasuhiro Iba

**Affiliations:** 1 School of Biological Sciences, Swire Institute of Marine Science, and Department of Earth Sciences, the University of Hong Kong, Kadoorie Biological Sciences Building, Pokfulam Road, Hong Kong, SAR China; 2 Department of Paleobiology, National Museum of Natural History, Smithsonian Institution, P.O. Box 37012, MRC 121, Washington, DC 20013-7012 USA; 3 BugWare, Inc., 1615 Village Square Blvd., Suite 8, Tallahassee, Florida 32309 USA; 4 Department of Earth and Planetary Sciences, Hokkaido University, N10W8, Kita-ku, Sapporo, Hokkaido, 060-0810 Japan

## Abstract

The Cretaceous Period stands out in Earth’s geologic history by ubiquitous and sustained massive eruption of lava, forming several enormous igneous plateaus in the ocean basins worldwide. It has been proposed that the subaerial phases of Cretaceous oceanic plateau formation spurred the global environmental deterioration, yet this view is supported by patchy fossil and/or rock evidence for uplifting of the plateau summits above the sea level. Reported here is by far the most comprehensive case of Cretaceous plateau emergence at northern Shatsky Rise, Northwest Pacific, based on the integration of unique micropalaeontological and seismic evidence. From just above the flat-topped igneous edifice, recent Integrated Ocean Drilling Program (at Site U1346) recovered early Cretaceous (Hauterivian) ostracod and foraminiferal assemblages showing marked shallow-marine preferences. Most intriguing discovery is an ostracod taxon with well-developed eye tubercles, which serves as compelling palaeobiological evidence for a very shallow, euphotic setting. By linking the nearshore biofacies (<20 m water depth) to the basement topography undoubtedly shaped by subaerial weathering and/or erosion, it is obvious that northern Shatsky Rise was remarkably emergent during its final emplacement phase. We suggest that early Cretaceous surface environments might have been affected, at least in part, by Shatsky Rise subaerial volcanism.

## Introduction

The mechanism of formation of massive basaltic plateaus (or large igneous provinces [LIPs]) in time and space has been one of highly intriguing topics in solid-earth geoscience since the early 1990’s when the mantle plume hypothesis became popular^1^. It has also become an important key to understand the evolution of Earth’s biosphere because such volcanic pulses might have been the fundamental drivers of past major environmental deterioration and biotic crises^2,3^. The LIP activity was at its zenith in the oceans during the Cretaceous Period^2,4^, and its global environmental impacts might have been dependent on the lava eruption depth relative to the sea level. If subaerial, emissions of CO_2_, SO_2_, halogens, among others, from oceanic LIPs could have directly impacted both marine and terrestrial Cretaceous ecosystems by the greenhouse effect and the spread of acidic substances^2,3,5,6^. In addition, there is growing evidence for the role of subaerial weathering of basalts and volcaniclastic material on ocean iron fertilization^7^, the process that can cause enhanced primary production and, possibly, ocean anoxia and mass extinction.

**Figure 1:**
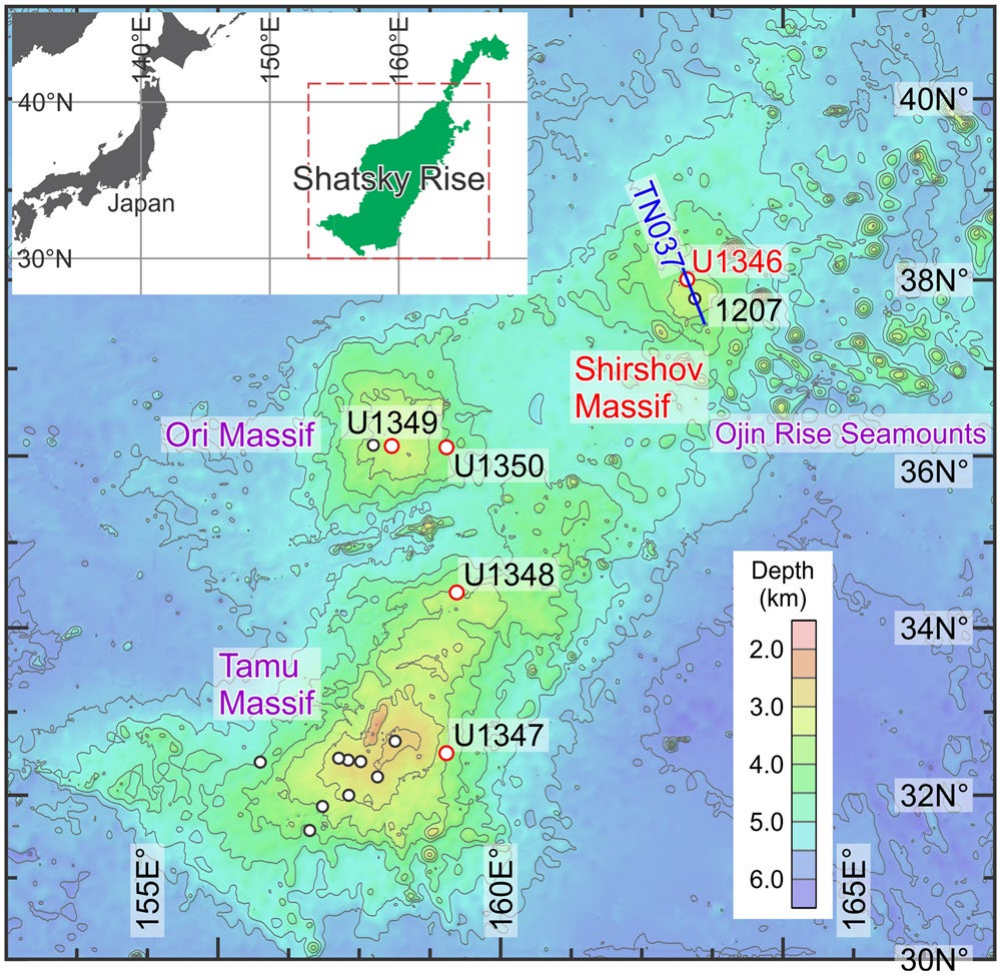
Bathymetric map of Shatsky Rise, Northwest Pacific. Locations of scientific drilling are indicated for Integrated Ocean Drilling Program Expedition 324 (Sites U1346–U1350: red circles) as well as for ODP Site 1207 and other previous cruises (open circles). TN037 (blue) indicates line of seismic cross-section in Fig. 3. Base colour seafloor map is SRTM30_PLUS and SRTM15_PLUS Global Bathymetry grid developed by SIO, NOAA, U.S. Navy, NGA, and GEBCO^37,38^ (http://topex.ucsd.edu/WWW_html/mar_topo.html). Inset map (top left) is from ref.^39^ (credit to source image—Ando, A. *et al*. 2016. An emerging palaeoceanographic ‘missing link’: multidisciplinary study of rarely recovered parts of deep-sea Santonian–Campanian transition from Shatsky Rise. *figshare*, https://doi.org/10.6084/m9.figshare.3453269.v1, used after modification under a Creative Commons Attribution 4.0 International Public License [CC BY 4.0]).

Lying 3 to 5 km beneath the Northwest Pacific sea surface, Shatsky Rise is a deep-marine igneous plateau formed through the latest Jurassic–early Cretaceous Pacific LIP volcanism, with a total area as large as Japan^8,9^ (Fig. 1). Due to rarity of such an old oceanic rock record, Shatsky Rise has long been a particularly attractive target of marine geological studies since 1960’s. Despite repeated scientific drilling campaigns, past drill holes seldom reached the igneous basement, and so little information was extracted on the birth and emplacement of this massive oceanic plateau. The poorly constrained early history of vertical tectonics and palaeobathymetry, with only tenuous clues from dredged limestones^8^, has been a major obstacle in interpreting the aforementioned palaeoenvironmental significance of the Shatsky Rise volcanism. In 2009, Integrated Ocean Drilling Program (IODP) Expedition 324 focused primarily on basement drilling of Shatsky Rise, recovering a wealth of igneous-rock core samples for multidisciplinary study^9,10^. A foregoing report on geochemistry of fresh volcanic glasses^11^ was successful in generating specific estimates on the eruption depths for two out of three major volcanic edifices, Tamu and Ori Massifs (Fig. 1), and shed new light on vertical tectonics of Shatsky Rise in its infancy. However, Shirshov Massif (Fig. 1) has remained nearly unexplored by such a geochemical technique because of pervasive rock alteration. To elucidate the early palaeo-elevation history and to address the potential environmental impacts of this Cretaceous oceanic LIP, here we present new fossil evidence from sedimentary rocks atop the basement of this underexplored northern Shatsky Rise region.

**Figure 2:**
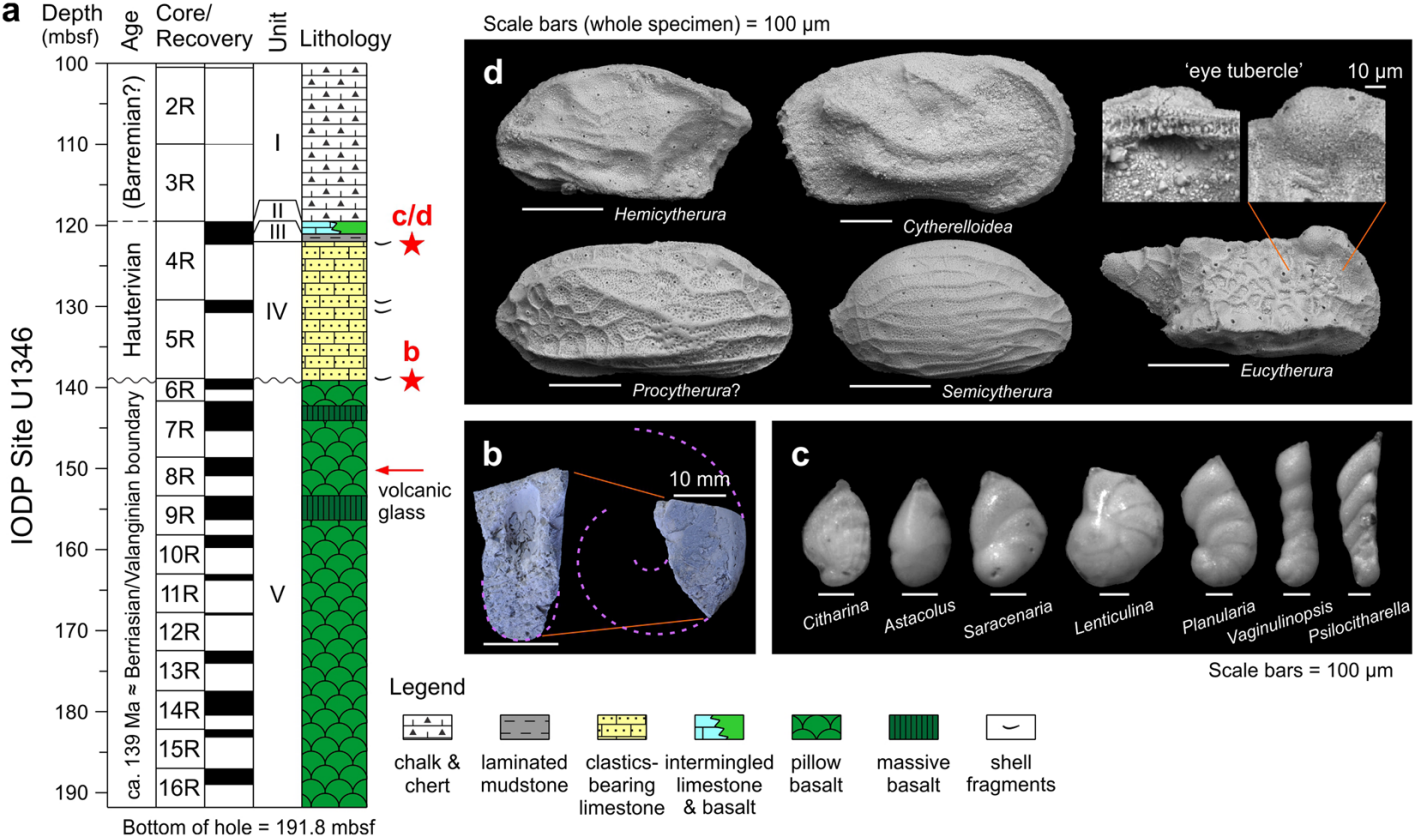
New rock and fossil records of Integrated Ocean Drilling Program (IODP) Site U1346, Shirshov Massif, northern Shatsky Rise. (**a**) Summary lithostratigraphy^12^ with symbols indicating key fossil occurrences (stars) and fresh volcanic glass (arrow; analyzed at single level^11^). For age interpretation see Methods. mbsf—meters below seafloor. (**b**) Ammonite (Desmoceratidae gen. & sp. indet.) from just above igneous basement; right lateral (right) and cross-section (left) views of incomplete specimen, with broken lines delineating inferred lost part of whorl (see also Supplementary Fig. S1). (**c**,**d**) Microscopic images of key representative genera of benthic foraminifera (**c**) and ostracods (**d**) from two samples 324-U1346A-4R-CC, 3–4 cm and 7–8 cm. Note paired magnified images of eye tubercle on *Eucytherura*, viewed from valve exterior (right) and interior (left).

Shirshov Massif was drilled at IODP Site U1346 (Fig. 1), for which pre-cruise seismic survey illustrated a thin sediment cover blanketing the flank of this volcanic edifice, thereby assuring the ease of access to the basaltic basement. Site selection at this peripheral area of the plateau, eventually, led to unprecedented opportunity to study the conspicuous sedimentary record^12^ that had not been encountered through the history of Shatsky Rise drilling. The unique sedimentary interval is from Cores 6 R (part) to 4 R and comprising, in ascending order, limestones rich in clastics and molluscan bioclasts (Unit IV), rhythmically-bedded siltstone with minor turbiditic sandstones (Unit III), and intermingled limestones and basalts (Unit II) (Fig. 2a). Overall, it should represent a deepening-upward marine sequence, from more or less neritic sediments (Unit IV) to intermediate, gravitational clastic/calcareous deposits (Unit III/II) to deep, fully pelagic chalk/chert (Unit I). A notable find among macrofossils is the Desmoceratidae ammonite from near the base of Unit IV, just above the basaltic basement (Fig. 2b; Supplementary Fig. S1). Microfossils are present through Unit IV to II, including moderately/poorly preserved nannofossils indicative of the Hauterivian Age^12^, whereas planktonic foraminifera are notably lacking (for additional notes see Methods). Remarkably, and as detailed below, a variety of well-preserved benthic foraminifera and ostracods have been retrieved from a thin, semi-consolidated marly interval in the uppermost Unit IV.

## Results

The benthic foraminiferal and ostracod assemblages can be considered as reliable indicators of the depositional water depth of the Shirshov Massif sediments. The former comprises 10 genera of the Family Nodosariidae/Vaginulinidae (*Astacolus*, *Citharina*, *Dentalina*, *Frondicularia*, *Laevidentalina*, *Lenticulina*, *Planularia*, *Psilocitharella*, *Saracenaria*, *Vaginulinopsis*)^12^ (Fig. 2c). The nodosariid/vaginulinid benthic foraminfiera occur mainly from the neritic setting and often as deep as the upper bathyal depth^13,14^, yet such a monofamily assemblage without typical deep-water taxa is indicative of the neritic environment.

A narrower range of palaeo-water depth, probably in a coastal inner neritic setting shallower than 20 m, is estimated from the ostracod assemblage with 6 genera (*Cytherelloidea*, *Eucytherura*, *Hemicytherura*, *Paracypris*?, *Procytherura*? *Semicytherura*) of potentially up to 12 species (Fig. 2d). Following are the key features that support reconstruction of such nearshore marine environment: (i) assemblage composition dominated by the Family Cytheruridae and with *Cytherelloidea*, resembling a Jurassic Argentine inner shelf biofacies^15,16^; (ii) abundant occurrence of known seaweed-associated intertidal taxa, *Semicytherura* and *Hemicytherura* ^17–19^; (iii) generally low species-diversity^20^; and (iv) absence of typical outer neritic or deeper taxa such as *Cytheropteron*, *Argilloecia*, trachyleberidids and thaerocytherids (their absence is indicative of shallow [<20 m] depth in specific examples of modern western Pacific coasts)^21–23^.

Additionally, and most importantly, we document the common presence of large eye tubercles on *Eucytherura* (Fig. 2d). Eye tubercles, usually possessed by particular groups of post-Palaeozoic eurybathic ostracods, are larger in the shallow-marine counterparts, and are smaller with increasing water depth, eventually disappearing in habitats of middle bathyal or deeper depth^24–26^. The development of eye tubercles up to ~50 μm in diameter (like that shown in Fig. 2d) is an independent line of functional morphological or palaeobiological evidence that can definitely demonstrate the shallow, euphotic environment^24,25^. Ostracod water-depth reconstruction reinforced by such a known environmentally regulated functional determinant is highly effective rather than relying only on the aforementioned interpretation from comparative autoecology, especially with respect to extant taxa (modern analogue technique). That is, the latter approach is empirical and may be compromised by benthic habitat shifts that can occur through the course of macroevolution on a geologic time scale.

## Discussion

**Figure 3:**
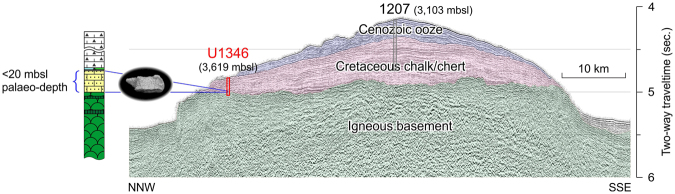
NNW–SSE seismic profile of Shirshov Massif along TN037 ship track from ref.^27^ (see also Fig. 1), with sub-seafloor geological interpretation of igneous basement, Cretaceous chalk/chert, and Cenozoic ooze^12,40^. mbsl—meters below sea level.

With new definitive fossil evidence for near-sea level (<20 m at the shallowest) for deposition of Site U1346 sediments, the flat-topped acoustic basement of Shirshov Massif ^8,27^ (Fig. 3) can be interpreted as a topographic expression of subaerial/wave erosion. Taking a closer look at, the summit flatness is distorted to a minor extent (and to a larger extent in another orthogonal seismic profile^27^), but this feature is explained by later tectonic modifications that accompanied ubiquitous small-scale intrusions, namely the Ojin Rise Seamounts^8^ (Fig. 1). Meanwhile, it is noteworthy that a flat basement top is also seismically imaged for a part of Ori Massif ^8^, and that cores just above the Ori Massif basement (IODP Site 1349) have been found to contain some sedimentary components indicating subaerial erosion as well as deposition near the sea level^9,10^. Altogether, the Shatsky Rise volcanism is understood to have occurred subaerially in the northern region, and possibly in the central region as well, during the final phase of the plateau emplacement.

Interestingly, our micropalaeontological evidence for the plateau emergence does not exactly match the independent eruption-depth reconstructions at the same site by means of lava vesicularity and volcanic glass CO_2_-H_2_O concentrations (Fig. 2a), both of which give somewhat deeper estimates (with errors) of <300 m and 700–800 m below sea level, respectively^9,11^. This discrepancy in estimated palaeo-depths between the sediment and basement sections would be accounted for by a temporarily accelerated final vertical tectonics, uplifting the young, initially submarine plateau to the elevation well above the sea level in a geologic instant.

Revelation of the early emergent phase of Shatsky Rise suggests that this oceanic LIP had a potential of effectively impacting early Cretaceous biogeochemical cycles via direct emissions of CO_2_, SO_2_, halogens, as well as vigorous weathering of basalts and volcaniclastics^2,3,5–7^. Based on available Site U1346 age data (see Methods), though not definitive, the subaerial volcanism and summit weathering/erosion occurred during the Valanginian. This period is well-known for major carbon cycle perturbations, such as enhanced marine carbon burial, global δ^13^C shift, and demise of carbonate platforms^28–30^. The primary trigger of the Valanginian palaeoenvironmental events has been considered by many as the warming induced by the Paraná-Etendeka LIP volcanism. This view is supported by Pb isotopic fingerprinting^31^, but unsupported by radiometric dating^28^. Therefore, it may be worthwhile exploring the involvement of Shatsky Rise subaerial volcanism to the Valanginian events. If substantiated, it can explain some of the palaeoenvironmental episodes, including possible anoxic condition in the Pacific^29^, at that time.

The definitive case history of emergence of Shatsky Rise adds confidence to the universality of this geologically outstanding phenomenon. Major Cretaceous oceanic plateaus of Ontong Java, Manihiki, Kerguelen, and Caribbean have already been known for the physical (fossil and/or rock), albeit patchy, evidence for their past uplifting more or less above the sea level^4,5,32,33^; some developed discussions on their palaeoenvironmental consequences by assuming the massive subaerial volcanism of these plateaus^5,6^. However, these attempts were not so convincing because none of those oceanic LIPs retain the topographic expressions of large-scale emergence due to later tectonic disturbance of the original plateau geomorphology. Therefore, our combined fossil-seismic evidence from Shatsky Rise is crucial in making reasonable inference, that significant early emergent phases would have been fundamental to Cretaceous oceanic plateaus. It will also help facilitate the study of surface interaction of solid-earth and biogeochemical processes in understanding Earth’s Cretaceous palaeoenvironmental evolution.

## Methods

### Micropalaeontology

For separation of fossil foraminifera and ostracods, sediment samples (see Fig. 2 caption) were treated in dilute hydrogen peroxide, wet-sieved, dried, and examined under a stereomicroscope at >125 μm fraction. Scanning electron microscopic imaging was performed at the SEM Lab, National Museum of Natural History, Smithsonian Institution. Detailed palaeontological description of the microfossils encountered will be reported elsewhere.

It is noteworthy that planktonic foraminifera are completely absent in the examined samples, and such observation may also be attributed to a very shallow bathymetric setting. However, during the early Early Cretaceous (Hauterivian and older), planktonic foraminifera were in the course of early evolution with restricted geographic distribution at epicontinental seas as well as small sizes and very low diversity^34^. These palaeobiogeographic and evolutionary contexts more reasonably explain the non-existence of planktonic foraminifera of this age at Site U1346 and other Shatsky Rise sites in the then central Pacific.

### Age model

Age assignments for the basement and sediment sections of Shirshov Massif are based on the following observations and assumptions. The age of the basement rocks is near the Berriasian/Valanginian boundary (~139 Ma), considering the seafloor magnetic lineation of Chron M13-14^9,10^. Such dating based on magnetic lineation can be validated by recent ^40^Ar/^39^Ar geochronology for Tamu Massif (ref.^35^), which demonstrated consistency in age data between the seafloor magnetic anomalies and the actual radiometric measurements. A Hauterivian age for a part of the sedimentary interval is based on nannofossils obtained from Cores 5 R and 4R^12^. Overlying chalk/chert interval of Cores 3 R to 1 W was not dated directly using microfossils (due to rotary core drilling that disintegrated softer chalk sediments, leaving chert nodules only), but it is presumably of Barremian age in light of seismic correlation between ODP Site 1207 (Fig. 3). Of note is the presence of the Desmoceratidae ammonite (total range: late Valanginian–Maastrichtian)^36^ (Fig. 2b; Supplementary Fig. S1) from the basal sedimentary unit of Core 6R-1, which can indicate, at least, that sediments as old as the early Valanginian do not exist. In summary, it is probable that the basement/sediment contact is represented by an unconformity due to subaerial erosion erasing several million years of the Valanginian interval.

## Electronic supplementary material

Supplementary Figure
