# Supplementary material for: Past emergent phase of Shatsky Rise deep-marine igneous plateau
Source: Sci Rep. 2017 Nov 13;7:15423. doi: 10.1038/s41598-017-15684-z (PMC5684222; doi:10.1038/s41598-017-15684-z)
Supplement: Supplementary file 1 — Supplementary Figure [file 41598_2017_15684_MOESM1_ESM.pdf]

Supplementary Information [Manuscript # SREP-17-35867]

Past emergent phase of Shatsky Rise deep-marine igneous plateau

Moriaki Yasuhara, Atsushi Ando, and Yasuhiro Iba

Supplementary Figure S1

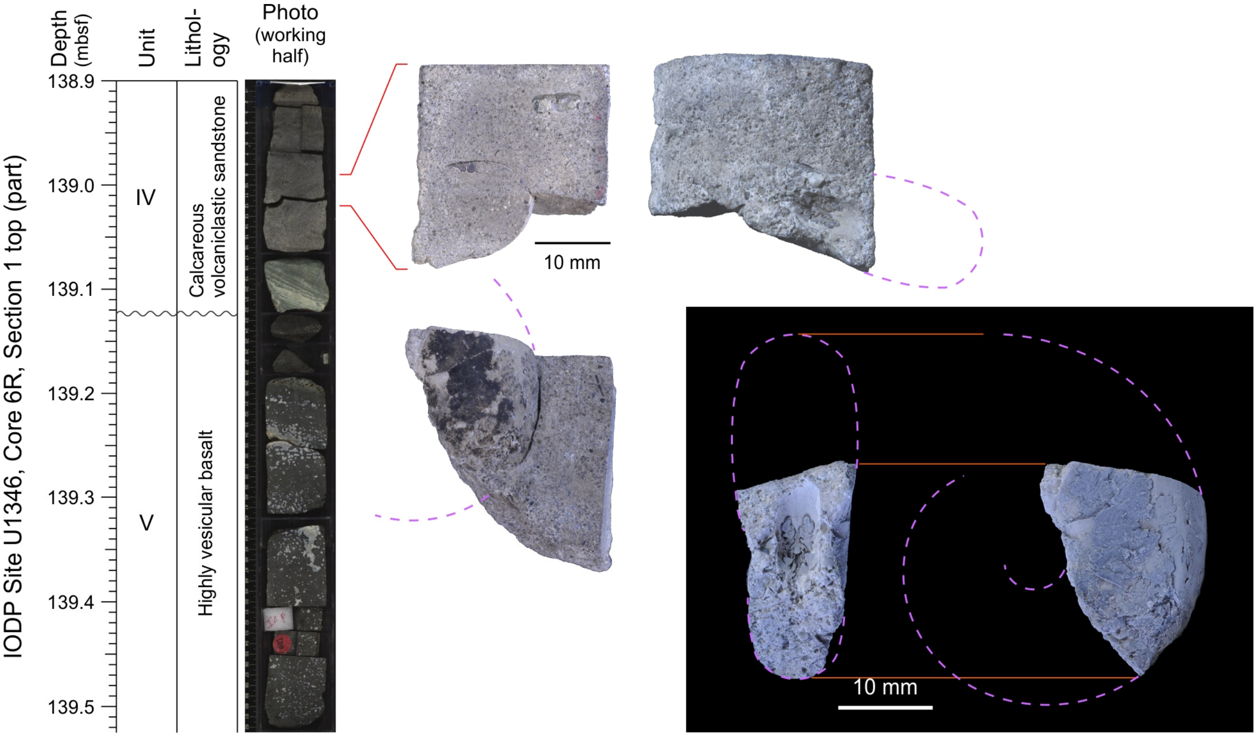

Ammonite Desmoceratidae gen. & sp. indet. fortuitously found from just above the igneous basement of IODP Site U1346 (interval 324-U1346A-6R-1, 10–12 cm). This fragmented specimen is deposited in the National Museum of Nature and Science, Tokyo (collection number: NMNS PM 35015), without separation from the host rock, as shown. The paired images at bottom-right (also Fig. 2b in main text) are right lateral and cross-section views photographed after whitening (digitally cropped). Broken lines delineate inferred missing part of the whorl. The following features illustrate affiliation to the Family Desmoceratidae (Wright, 1996): (i) a medium-narrow umbilicus; (ii) relatively compressed whorl section; (iii) broadly rounded venter; (iv) slightly convex flank; (v) highly subdivided suture with deep tripartite lateral lobes; and (vi) narrow and deep internal lobe.
